# Enlargement of female pupils when perceiving something cute

**DOI:** 10.1038/s41598-021-02852-5

**Published:** 2021-12-03

**Authors:** Kana Kuraguchi, Kei Kanari

**Affiliations:** 1 Graduate School of Human Sciences, Osaka University, 1-2 Yamadaoka, Suita City, Osaka 565-0871 Japan; 2 Faculty of Psychology, Otemon Gakuin University, 2-1-15 Nishiai, Ibaraki City, Osaka 567-8502 Japan; 3 Department of Fundamental Engineering, Utsunomiya University, 7-1-2 Yoto, Utsunomiya, Tochigi 321-8585 Japan

**Keywords:** Psychology, Human behaviour

## Abstract

It is reported that women’s pupils dilate when they see a baby; it is unclear if this pupillary response is caused by the perception of cuteness itself. Since many objects besides babies can be perceived as cute, we investigated whether the perception of cuteness, or the type of object observed, is related to pupil dilation. In the first experiment, female participants were requested to rate the subjective cuteness of greyscale pictures of objects such as animals and foods; their pupil sizes were measured. The results showed a significant positive correlation between perceived cuteness and participants’ pupil dilation. In the second experiment, participants rated the cuteness of images of female faces. Results revealed a significant negative correlation between perceived cuteness and pupil dilation. In our study, perceiving cuteness enlarged female observers’ pupils except when observing female faces. Positive reactions associated with cuteness may be premised on the existence of unconscious perceptual alterations and physical responses.

## Introduction

Perceiving cuteness encourages us to approach the cute object and provides motivation for caretaking^1^. Additionally, perceiving cuteness narrows our perceptual attention^2^ and makes it difficult to compare two faces in our peripheral vision^3^. These findings show that perceiving or judging cuteness focuses our attention on the cute object and suggest that cuteness evokes interest in the object to facilitate communication. In other words, when trying to judge cuteness, there may be a top-down change in perception.

Previous studies have revealed that female reproductive hormones have an effect on sensitivity in perceiving cuteness^4,5^. Furthermore, it has been shown that in general women are more attuned to perceiving cuteness^5–9^. From the perspective of evolutionary psychology, cuteness motivates us to raise a child. In most cases, women play the role of primary caregiver, which seems to be consistent with the finding that women are more sensitive to cuteness. Hess^10^ revealed that an observer’s pupil size enlarges when looking at what interests them: women’s pupils dilate when looking at photographs of babies or male models, while men’s pupils dilate when looking at female models. The results of the pupil measurements also suggest that women may be more interested in babies, which may reflect differences in sensitivity to cuteness. Since babies induce us to perceive cuteness, cuteness perception itself may cause the pupil to dilate, especially for women. However, this point has not been investigated previously. Aesthetic or attractiveness judgments are related to cuteness, and previous studies have shown that these judgements are correlated with changes in pupil diameter. For example, in a study which used faces as stimuli, the pupils of participants dilated as ratings of aesthetic pleasantness increased^11^ and contracted as ratings of attractiveness increased^12^. Pupillary response is also associated with uncanny, which is considered to be the opposite of cute, and weaker pupillary dilation has been shown for uncanny faces^13^. Therefore, the level of cuteness may also influence pupil response. On the other hand, cuteness has been differentiated from attractiveness and beauty^14,15^, and different visual responses to cuteness have been shown even in response to the same image^3,14^. By using the pupillary response as an unconscious physical reaction, we may clarify the differences and similarities in the axes of judgments of cuteness and aesthetic pleasure or attractiveness.

Perception of cuteness is produced by baby schema: a set of physical features possessed by infants, such as large eyes, a small mouth, chubby cheeks, and a high and protruding forehead^16^. The physical characteristics babies and young animals typically have represents this baby schema. Previous studies have shown that the more consistent infants’ faces are with the schema, the cuter they are thought to be^1,5,8,17,18^. It is also known that babies with faces that are more consistent with the baby schema are looked at for longer^19–21^. However, cuteness is not only felt in response to babies, but also to adults^3,22^. We also feel a sense of cuteness about objects rather than people. For examples, cuteness can be perceived not only in response to babies, images showing things such as desserts, accessories, and plants can also make us smile^23^. Also, factors affecting one’s perception of cuteness include not only types of objects but also shapes, such as circles, and colors, such as reddish hue^24^. In other words, the perceived object of cuteness may not be limited to those with baby schemas, such as babies. In this study, to clarify whether the perception of cuteness itself causes the pupil response, we analyzed the relationship between pupillary response and perceived cuteness using photographs of animals, food, and plants in Experiment 1, and adult female faces in Experiment 2. Since previous studies have shown that emotional arousal causes pupil dilation^25–27^, we used a neutral facial expression for all adult female faces.

In this study, we investigated whether subjective judgement of cuteness correlates with pupillary response and whether the correlation between the rating and the pupillary response is consistent for human faces and non-face images such as animals. If attempting to judge cuteness itself affects our perceptions, pupil dilation would occur just as it does when we see a baby, which automatically elicits cuteness, regardless of the observed object. In addition, the degree of pupil dilation may be greater as subjective cuteness increases. However, when the object to be evaluated is a face, emotions and information processing other than the perception of cuteness may be reflected. Therefore, there is a possibility that the results may differ between faces and other objects. For example, an unattractive face could be perceived as an indication that the person is unhealthy (e.g.,^15,28,29^), which may elicit an aversion or a feeling of disgust towards the person, resulting in a negative correlation between an observer’s pupillary change and perception of cuteness. Although men and women are equally motivated to look at cute babies^20^, women are more responsive to recognizing cuteness in a broader range of objects^9^. Since women are generally considered to be more sensitive to cuteness, this study included women as participants. Participants were asked to assess the cuteness of different objects, and we investigated whether the perception of cuteness produces a pupillary response.

## Experiment 1

### Methods

#### Participants

Twenty-two Japanese females between the ages of 18 and 24 years (mean age: 19.81 years), and who were unaware of the research’s purpose, participated in Experiment 1. All reported having visual acuity that was normal or corrected-to-normal and having no diagnosed neurological condition. All participants gave their written informed consent before the experiment began and were paid a reward according to the standard of Otemon Gakuin University.

#### Stimuli

**Figure 1:**
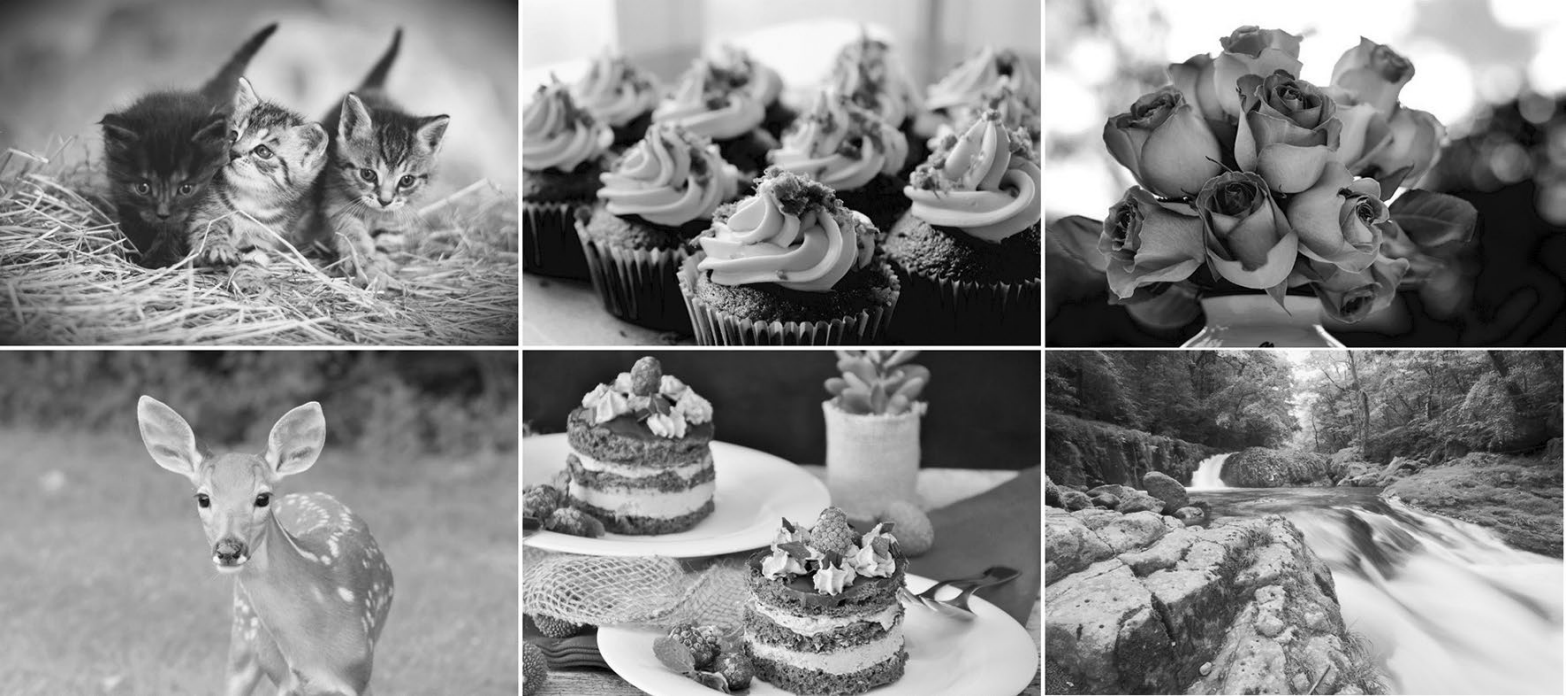
Examples of images presented in the experiment. Top left: ©congerdesign (Creative Commons); top middle: ©Free-Photos (Creative Commons); top right: ©jill111 (Creative Commons); bottom left: ©diane616 (Creative Commons); bottom middle: ©RitaE (Creative Commons); bottom right: ©Junkichi Egashira (Licenced under CC BY 4.0).

Forty greyscale images were used in this study. Original color images were obtained from the Internet. All images were royalty-free, Creative Commons Zero or licensed under CC BY 4.0. Images included pictures of animals, foods, flowers, and landscapes (Fig. 1). The number of images in each category varied: animals (21), food (6), flowers (4), landscapes (7), stuffed animals (1), and bags (1). These images were selected through the pre-evaluation (cuteness and pleasantness evaluation) of 39 individuals, and it was confirmed that cuteness and pleasantness were not correlated (*r* = 0.125, *p* = 0.442). The image subtended 35.1 × 23.3° (1280 × 853 pixels). Original color images were converted to greyscale using the “rgb2gray” function (NTSC standard) in MATLAB (MathWorks, Inc.). The pixel intensity of each greyscale image was normalized, using the formula *I*_*norm,i*_ = (*I*_*i*_ − *I*_*min*_)/(*I*_*max*_ − *I*_*min*_), where *I*_*norm,i*_ is the normalized pixel intensity, *I*_*i*_ is the original intensity, *I*_*max*_ is the maximum intensity, *I*_*min*_ is the minimum intensity. The mean luminance of the normalized image was manipulated with a gamma correction using the “imadjust” function in MATLAB so that the mean luminance fell between 20.0–20.5 cd/m^2^ (Output = Input^γ; adjusting γ). The mean luminance of all images was 20.15 cd/m^2^ (*SD* = 0.17, *Min* = 20.001, *Max* = 20.499). A luminance meter (LS-110, KONICA MINOLTA) was used to test the luminance of a grayscale circle (2°) presented at the center of the display when the RGB values of the circle was changed by 10 to estimate the luminance of the image from a lookup table.

#### Procedure

**Figure 2:**
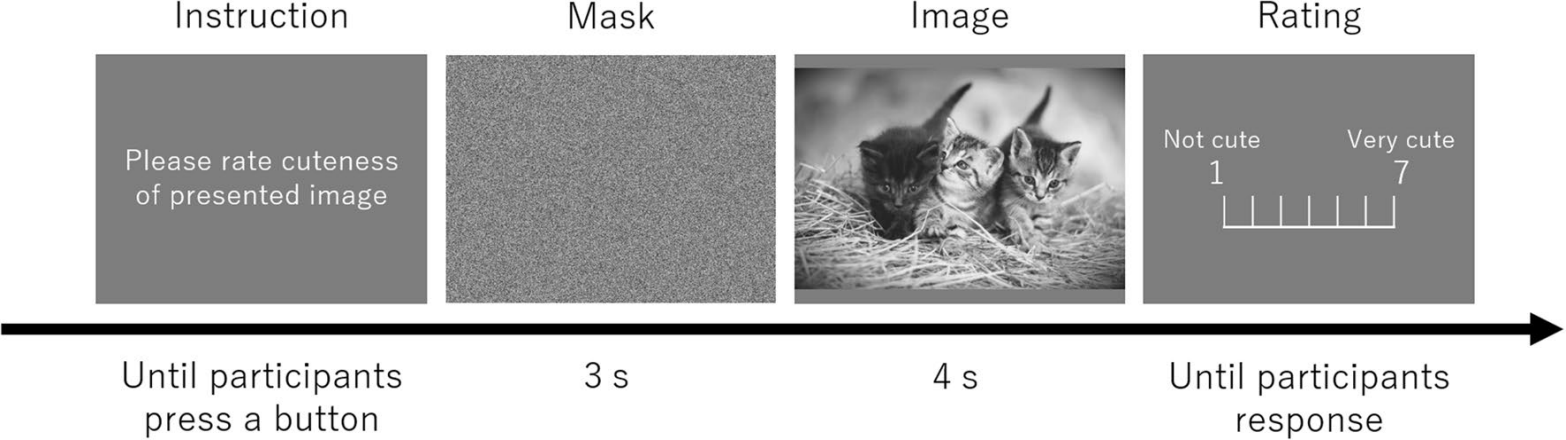
The procedure for a trial. Participants press any response key for rating, and then instruction appears again. Participants had to wait 3 s after pressing a response key and to press a button to start the next trial.

Each participant completed three blocks, representing 120 total trials. Each block had 40 randomly-presented trials. In other words, each participant observed each stimulus three times to ensure the accuracy of the pupil change measurement. This method has been used in previous studies (e.g.,^30,31^). In each trial, the participant was first instructed to rate the subjective cuteness of a stimulus that will be presented hereafter. After the participant pressed the button, a mask stimulus consisting of scrambled dots (20.0 cd/m^2^) was presented for 3 s. Then, a test stimulus was presented at the center of the display for 4 s. Participants rated subjective cuteness of the test stimulus using a 7-point scale, ranging from 1 = not cute to 7 = very cute, by using a numerical keypad after the test stimuli. Although the response time was unlimited, participants were asked to not think too deeply about their response. There was a 3 s delay before the participant could press the button again to launch the next trial. This sequence is shown in Fig. 2.

#### Apparatus

The stimulus was displayed on a 21 in. CRT (SONY GDM F500R, 1280 × 960 pixels, 35.1 × 28.9°, 36 × 29.4 cm), that was specifically designed for precise manipulation of luminance. The room used for the trials was dark, and participants observed the stimuli with their head fixed by a chin rest, at a visual distance was 57 cm. Stimuli were produced and displayed using a PC (MacBook Pro, Apple Inc.) with MATLAB (MathWorks, Inc.) and Psychophysics Toolbox extensions^32,33^.

#### Pupillometry

Pupil size was recorded using the EyeLink 1000 eye tracker (SR Research Ltd.) that had a sampling rate of 1000 Hz. Data for 150 milliseconds (ms) before and after the blinks were excluded from the analysis. Trials were excluded if data from the trial was missing in excess of 35%. Missing data were reconstructed using a cubic spline fit. The pupil size for 100 ms before the test stimulus presentation was averaged and used as the baseline. The pupil size during each trial’s 4 s presentation of the test stimulus was normalized as the rate of change from the baseline. Normalized pupil sizes were averaged across trials for each participant and stimulus.

### Results

**Figure 3:**
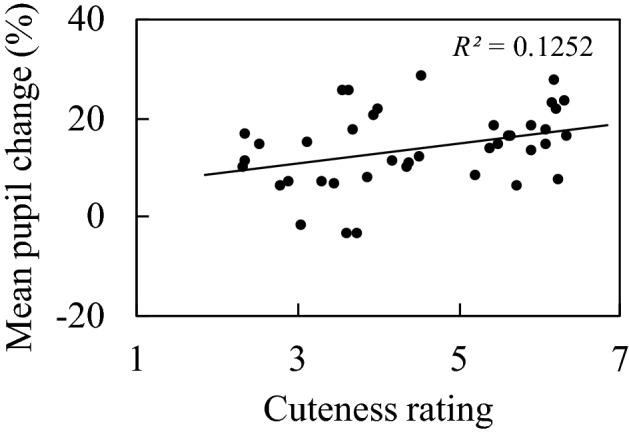
The correlation between the cuteness of the images and the mean pupil change. The value in the upper right corner of the panel indicates the coefficient of determination.

We investigated the correlation between rated cuteness of the image and the mean pupil change, which is shown in Fig. 3. The ordinate indicates the mean percentage change in pupil size between the baseline and the test stimulus (4 s). The abscissa presents the cuteness rating for the test stimulus. Each dot in Fig. 3 corresponds to a stimulus image, and mean percent change in pupil response and mean rating values for all participants were used in the correlation analysis. The correlation between the cuteness of the image and the mean pupil change was significant (*r* = 0.3538, *p* = 0.0250). The images were classified into either animal or non-animal categories, and the correlations between cuteness ratings and pupil changes in each category were analyzed. No significant correlations were obtained in any of the categories (animal: *r* = 0.299, *p* = 0.187; non-animal: *r* = 0.218, *p* = 0.370). Therefore, the following analysis was conducted on all images.

**Figure 4:**
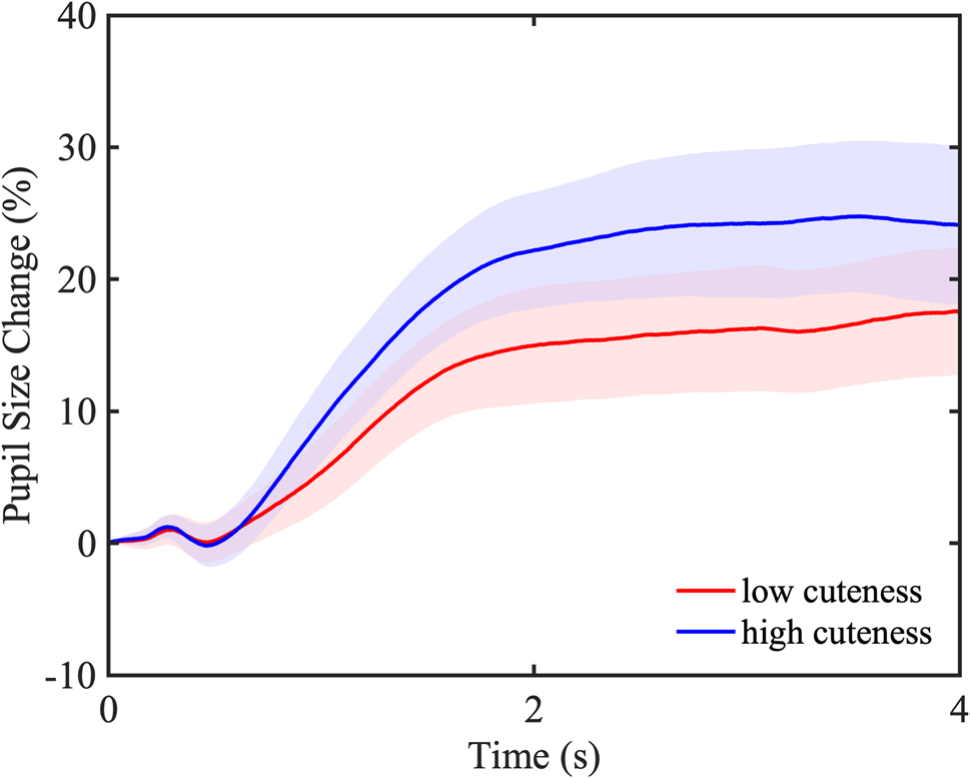
Dynamics of pupil change over the entire presentation time. The blue line shows the mean pupil change corresponding to the 20 images with a high cuteness rating, and the red line shows the mean pupil change corresponding to the 20 images with a low cuteness rating. The blue-colored area indicates a 95% confidence interval for the images with high cuteness rating, and the red-colored area indicates a 95% confidence interval for the images with low cuteness rating.

To show the dynamics of pupil change, we divided the stimulus images into high and low groups of 20 images each based on cuteness ratings and illustrated the mean pupil change over the entire presentation time (4 s) for each group in a time series (Fig. 4). Because the mask image with the same luminance as the stimulus image was presented for 3 s before the stimulus image, pupil constriction was hardly observed immediately after the presentation of the stimulus image. In addition, pupils dilated more in response to the cute images than to the less cute images throughout the presentation time.

We performed three types of luminance checks to investigate whether the luminance of images produces a spurious correlation between rated cuteness and the mean pupil change. First, the mean luminance and the standard deviation (SD) of luminance distribution of an image affect pupil response^34^. Therefore, we tested the correlation between these values and pupillary changes. The correlation between the mean luminance of the images and pupil change was not significant (*r* = 0.1285, *p* = 0.4294). However, the correlation between the SD of the luminance distribution and the pupil change was significant (*r* = 0.3850, *p* = 0.0142). Second, the luminance was distributed disproportionately between the upper and lower visual fields, and this may have strongly modulated the pupil response^35,36^. Therefore, we calculated the difference in luminance between the upper and lower parts of the image and examined whether the values differed between the high (cute) and low (less cute) groups. Additionally, we examined whether this difference correlated with pupillary change. Results showed that there was no significant difference between the high and low groups (*M*_*high*_ = 1.041 cd/m^2^, *M*_*low*_ =  − 1.255 cd/m^2^,*t*(38) = 0.734, *p* = 0.467). The correlation between the difference in luminance of the upper and lower parts of images and the pupillary change was not significant (*r* =  − 0.1182, *p* = 0.4673). Third, the gaze position of participants while observing the images was not restricted, and it is therefore possible that pupil responses differed depending on the position of the observer's viewpoint^37^. Therefore, we determined the correlation between the mean luminance of the fixation area and the pupillary response. Following the analysis method of Naber and Nakayama^38^, the display was divided into 36 × 27 bins (one bin: 0.975° × 1.070°), and the bin with the highest frequency of fixation was calculated for each image. The diameter of the fixation area was varied from 1° to 10°, and we examined whether the values differed between the high (cute) and low (less cute) groups, and whether it correlated with pupil change, for each eccentricity. There was no significant difference between the high and low groups at each eccentricity (19.234 < *M*_*high*_ < 19.898 cd/m^2^, 18.906 < *M*_*low*_ < 19.941 cd/m^2^, *t*(38) < 0.359, *p* > 0.722). The correlation between the mean luminance of the fixation area and pupillary change was significant when the eccentricity was between 1° and 6° (− 0.224 < *r* <  − 0.300, *p* > 0.060), but not when it was between 7° and 10° (− 0.348 < *r* <  − 0.456, *p* < 0.028). We then calculated partial correlation controlling four indicators of luminance, that is, the mean luminance of images, the SD of the luminance distribution, the difference in luminance between the upper and lower parts of the image, and the mean luminance of the fixation area at 10°. A significant partial correlation was found between the cuteness of the image and mean pupillary change (*r* = 0.497, *p* = 0.002).

Furthermore, pupil size can be modulated by saccade characteristics^39,40^. Therefore, we determined the correlation between the mean amplitude of the saccades and pupillary change to verify whether pupillary change was influenced by different eye movement patterns. Eye movements with an amplitude of 1° or more and a velocity of 35°/s or more were defined as saccades^39,40^, and the mean amplitude of the saccades in each image was calculated. The correlation between the mean amplitude of the saccades and the mean pupillary change was not significant (*r* = 0.0902, *p* = 0.5799). There was no significant difference in the mean amplitude of the saccades between the high and low groups (*M*_*high*_ = 5.678°, *M*_*low*_ = 6.193°, *t*(38) = 1.427, *p* = 0.162). We calculated partial correlation controlling the mean amplitude of the saccades in addition to the four indicators of luminance and found a significant partial correlation between the cuteness of the image and mean pupillary change (*r* = 0.538, *p* = 0.001).

## Experiment 2

### Methods

#### Participants

Fourteen Japanese females between the ages of 18 and 25 years (mean age: 20.71 years), who were unaware of the experiment’s purpose, participated in Experiment 2. Thirteen of these participants had participated in Experiment 1. All reported having visual acuity that was normal or corrected to normal, and had never been diagnosed with a neurological condition. All participants gave their informed, written consent before starting the experiment, and they were given rewards according to the standard of Otemon Gakuin University.

#### Stimuli

**Figure 5:**
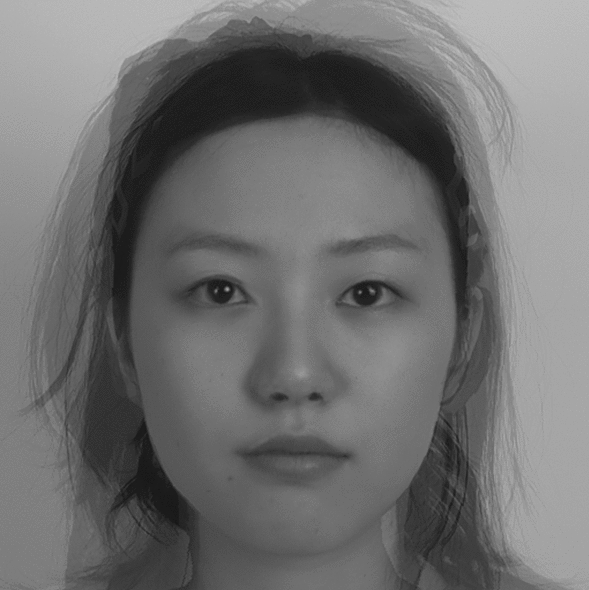
A sample of the face images used in Experiment 2. To protect model privacy, we show an average face created from a few of the face images used.

The stimuli consisted of facial images of 25 Japanese females (18–25 years, undergraduate and graduate students) with a frontal view and a neutral expression (Fig. 5). The stimulus subtended 14.0 × 14.0° (512 × 512 pixels). The images were converted to greyscale and normalized, and mean luminance was manipulated the same way as in Experiment 1. The mean luminance of all stimuli was 20.16 cd/m^2^ (*SD* = 0.15, *Min* = 20.006, *Max* = 20.489).

#### Procedure

The procedure used was the same as that of Experiment 1. However, the presentation of the fixation points and number of trials differed. For the presentation of the fixation point, a red fixation point (0.35°) was presented in the center of the mask (20.0 cd/m^2^, 14.0 × 14.0°) that was the same size as the stimulus because the stimuli were presented in the center of the display and were small relative to the display. Twenty-five face images were presented three times each (as in Experiment 1), adding up to 75 trials in total.

#### Apparatus and pupillometry

The stimulus presentation apparatuses for this experiment were the same as those used in Experiment 1. Additionally, pupil size was measured with the same apparatus used in Experiment 1 and analyzed in the same way, as well.

### Results

**Figure 6:**
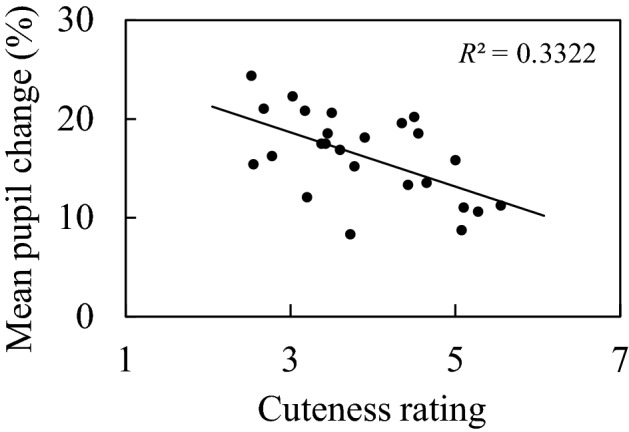
The correlation between the cuteness of the facial images and the mean pupil change. The value in the upper right corner of the panel is the coefficient of determination.

We examined the correlation between rated cuteness of the facial images and mean pupil change, which is shown in Fig. 6. Each dot in Fig. 6 corresponds to a stimulus image, and the mean percent change in pupil response and mean rating values for all participants were used in the correlation analysis. The correlation between the cuteness of the image and the mean pupil change was significant (*r* =  − 0.576, *p* = 0.0016).

**Figure 7:**
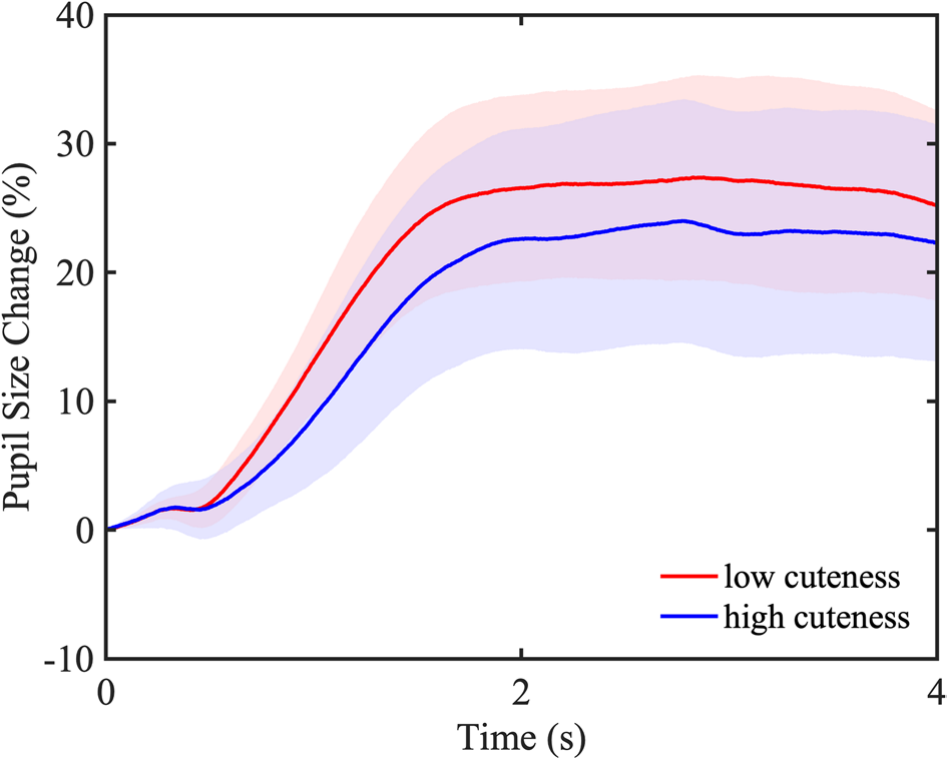
Dynamics of pupil change over the entire presentation time. The blue line shows the mean pupil change of the 12 images with a high cuteness rating, and the red line shows the mean pupil change of the 12 images with a low cuteness rating. The blue-colored area indicates a 95% confidence interval for the images with high cuteness rating, and the red-colored area indicates a 95% confidence interval for the images with low cuteness rating.

To show the dynamics of pupil change in Experiment 2, we divided the stimulus images into high and low groups of 12 images each based on cuteness ratings and illustrated the mean pupil change over the entire presentation time (4 s) for each group in a time series (Fig. 7). Also in Experiment 2, pupil constriction was hardly observed immediately after the presentation of the stimulus image, because the mask image with the same luminance as the stimulus image was presented for 3 s before the stimulus image. We also observed more pupil dilation throughout the presentation time for the images of less cute faces than for the images of cute faces in Experiment 2, which was different from Experiment 1.

We performed three types of luminance checks, as in Experiment 1. The correlations between the mean luminance of the images and the pupil change (*r* = 0.4246, *p* = 0.0344), and between the SD of the luminance distribution and pupil change (*r* = 0.5420, *p* = 0.0051) were significant. For the luminance difference between the upper and lower parts of images, the correlation with pupil change was not significant (*r* =  − 0.0029, *p* = 0.9890). There was no significant difference between the high and low groups (*M*_*high*_ =  − 5.597 cd/m^2^, *M*_*low*_ =  − 5.988 cd/m^2^; *t*(22) = 0.291, *p* = 0.774). For the mean luminance of the fixation area, the correlations with pupil change were not significant at all eccentricities (1° to 10°: − 0.008 < *r* < 0.192, *p* > 0.358). There was no significant difference between the high and low groups at each eccentricity (7.193 < *M*_*high*_ < 9.986 cd/m^2^, 5.861 < *M*_*low*_ < 10.717 cd/m^2^; *t*(22) < 0.953, *p* > 0.351). We also calculated partial correlation controlling four indicators of luminance, that is, the mean luminance of images, the SD of the luminance distribution, the difference in luminance between the upper and lower parts of the image, and the mean luminance of the fixation area at 10°; we found a significant partial correlation between the cuteness of the image and mean pupil change (*r* =  − 0.477, *p* = 0.029).

We also examined the correlation between the mean amplitude of the saccades and mean pupil change, as in Experiment 1, and found a significant correlation (*r* =  − 0.553, *p* = 0.004). There was no significant difference between the high and low groups (*M*_*high*_ = 1.865°, *M*_*low*_ = 1.801°; *t*(22) = 0.953, *p* = 0.351). We calculated partial correlation, controlling the amplitude of the saccades in addition to the four indicators of luminance, and found a significant partial correlation between the cuteness of the image and the mean pupil change (*r* =  − 0.456, *p* = 0.043).

### Ethics statement

This study was approved by the ethics committee of Otemon Gakuin University (Approved Number 2019-15) and was conducted in accordance with the Code of Ethics of the World Medical Association (Declaration of Helsinki).

## Discussion

We investigated the relationship between perceived cuteness and pupillary response when observing and rating images. In Experiment 1, we found a significant positive correlation between perceived cuteness for non-human images and change in pupil size, which suggests that perceiving cuteness enlarges the pupil. In contrast, in Experiment 2, we found a significant negative correlation between perceived cuteness for female face images and change in pupil size, which suggests that the pupil is enlarged when observing less cute faces in particular. These trends remained consistent in partial correlations controlling for the effect of image luminance, suggesting that cuteness ratings are associated with pupillary changes. It should be noted, however, that in both Experiments 1 and 2, pupils were generally dilated when participants observed the images while judging their cuteness. It is suggested that perceived cuteness of different assessment objects had the same effect on the pupil response as a whole. However, since there was not a positive correlation between the level of perceived cuteness and the degree of pupil dilation in response to the faces of adult women, it is possible that the evaluation of cuteness of adult female faces is qualitatively different from the evaluation of other objects including babies, animals, or food. In addition, the positive correlation in Experiment 1 is consistent with the findings of a previous study examining the relationship between aesthetic pleasure and pupillary response^11^. Further, the negative correlation in Experiment 2 is consistent with the findings of a previous study examining the relationship between facial attractiveness and pupillary response^12^. According to the results of Experiment 1 and 2, the pictures of human faces cause the opposite pupillary response to that of other stimuli. We suggest some possible reasons for this: one is the effect of disgust at less cute faces, another is the effect of jealousy on the observers of the same gender and generation as the people photographed, and a third is the effect of subjective brightness of facial images. We discuss these effects below, but recommend that these effects should be examined in further empirical experiments.

First, less cute faces may trigger negative emotions in the observer, which in turn may increase pupil dilation. In short, a low-cuteness level indicates a low degree of adaptation, which may have prompted avoidance from the less cute faces. Cuteness is considered to be one of the aspects of attractiveness, and it is known that attractive facial features are used to evaluate cuteness in the adult female faces. Kuraguchi et al.^22^ revealed that cuteness ratings correlated strongly with attractiveness ratings, as there are only minor differences in the facial features (e.g., eye shape) used to make cuteness and attractiveness ratings. Therefore, other attractive facial features may also be used to assess cuteness. For instance, one of the features of attractive faces is symmetry, which is an index of health and quality of a mate (e.g.,^28,29^). If cute faces are symmetrical, less cute faces are asymmetrical, which reflects poor physiological health^29^. The pupil dilation of the observers in this study may have been caused by the disgust and aversion they felt in response to less cute faces.

Second, our participants were all females, so it is possible that emotions other than the perception of cuteness were evoked simultaneously when evaluating the cuteness of adult female faces. The cuteness rating itself was done in the same way for both types of evaluation targets (e.g., cat or female face) despite their differences, but feelings evoked by cuteness may be different depending on the evaluation target. For example, feelings evoked by seeing cuteness generally encourage approaching behaviour^1^, but might not have the same effect for cute faces belonging to the same gender and generation because there is no adaptive benefit in approaching them (e.g.,^41^). It is known that females pay special attention to how physically attractive other females are and experience jealousy^42,43^. Therefore, it is possible that jealousy disrupts the change in pupil size among female observers because they deem a human face of the same gender and generation as a rival. That is, perceiving high cuteness generally enlarges the pupil, as observed in Experiment 1, but might evoke a different response when rating the cuteness of a person of the same gender. Accordingly, the effect of jealousy may have inhibited the pupillary response of dilation for cuter faces in this study, which may have led to the negative correlation between perceived cuteness of female faces and pupil size change. This point should be investigated in future work.

Third, the subjective brightness of facial images may have affected pupillary response in Experiment 2. Previous studies have revealed that light skin is considered more attractive^44,45^. Additionally, increasing the contrast between the facial features and the skin enhances the femininity of an androgynous face^46^ and makes a female face more attractive^47^. Therefore, there may be a correlation between the appearance of lighter skin and a higher attractiveness rating. In this study, we found significant correlations between some indicators of luminance and pupillary changes, although we controlled for the mean luminance of facial images. However, we also found a significant partial correlation between cuteness and pupillary change when controlling the indicators of luminance. Therefore, the subjective brightness of facial images may play a role as an indicator of explanation for the correlation between cuteness and pupil change. Previous studies have revealed that the pupil responds to subjective brightness^48,49,38,50^. If subjective cuteness increases with the subjective brightness of facial images, the pupil size change should be small for cuter face images. Therefore, the change rate of pupil size might reflect both subjective cuteness and subjective brightness.

In the present study, the perception of cuteness led to an involuntary pupil response in which the pupil of the observer was dilated. Although this trend occurred regardless of the object being evaluated, the correlation between the cuteness rating and the magnitude of the pupil response differed between evaluation objects. For cuter objects, observers' pupils were generally more dilated, but for adult female faces, perceived cuteness was negatively correlated with the magnitude of the pupil response. One explanation for this difference is the emotional impact associated with cuteness perception^9,51^. In other words, the emotional states associated with the perception of cuteness may differ between the objects being evaluated, and these emotional states may be expressed as unconscious physical responses. Based on the finding that pupil dilation occurs when emotions are stimulated (regardless of whether they are pleasant or unpleasant)^25^, it may be said that emotional arousal is higher in response to adult female faces that are not cute, and to non-human images that are cute. This means that the emotional experience of cuteness differs between adult female faces and non-human images. In the present study, however, the adult female faces were standardized to be neutral in expression, and the pleasantness and unpleasantness ratings did not correlate with cuteness, even for non-human images. In addition, Nittono^9^ stated that the response to cuteness is defined by moderate arousal. Therefore, it is difficult to explain the differences observed in the present study solely in terms of differences in emotional arousal. However, the effects of cuteness and emotional arousal on unconscious physical responses should be examined in the future. The pupil responses shown in the present study may also reflect a change in the quality of cuteness perception depending on the object being evaluated. This study shows that unconscious physical reactions occur when judging cuteness, using pupil response as an indicator, and provides an explanation for the process of positive reactions (e.g., motivation to approach) associated with cuteness.

## Conclusion

The perception of cuteness enhances approach motivation and encourages nurturing behaviours, which may be premised on the existence of unconscious perceptual alterations and physical responses. In this study, we found that judgments of cuteness are accompanied by pupillary changes, which are unconscious physical responses. By focusing on pupil changes in women who are sensitive to cuteness, we found that the perception of cuteness itself, even though differences in the response to the object being evaluated, could be the overall cause of pupil dilation.
